# An LC Passive Wireless Gas Sensor Based on PANI/CNT Composite

**DOI:** 10.3390/s18093022

**Published:** 2018-09-10

**Authors:** Sanmin Shen, Zhihong Fan, Jiahao Deng, Xiaowei Guo, Lei Zhang, Guanyu Liu, Qiulin Tan, Jijun Xiong

**Affiliations:** 1 School of Mechatronical Engineering, Beijing Institute of Technology, Beijing 100081, China; shensanming@nuc.edu.cn (S.S.); bitdjh@bit.edu.cn (J.D.); 2 Key Laboratory of Instrumentation Science and Dynamic Measurement, Ministry of Education, North University of China, Taiyuan 030051, China; 18435131963@163.com (Z.F.); 18234157656@163.com (X.G.); 18734136023@163.com (L.Z.); lgyydd@163.com (G.L.); xiongjijun@nuc.edu.cn (J.X.); 3 Science and Technology on Electronic Test and Measurement Laboratory, North University of China, Tai Yuan 030051, China

**Keywords:** polyaniline, carbon nanotubes, wireless passive gas sensor, LC mutual coupling, gas sensitive materials, NH_3_

## Abstract

This paper proposes a wireless passive gas sensor based on the principle of LC mutual coupling. After the acidification of the carbon nanotube (CNT), the in-situ polymerization of the aminobenzene monomers was conducted on the surface of the acidified CNT to form a sensitive material composed of a polyaniline/carbon nanotube (PANI/CNT) composite. The Advanced Design System (ADS) software was used for simulating and analyzing the designed structure, which obtained the various parameters of the structure. A lead-free aluminum paste was printed on an alumina ceramic substrate via the screen printing technique to form an inductor coil, before the gas sensitive material was applied to prepare a wireless passive gas sensor, consisting of a single-turn inductor and interdigitated electrodes on the base structure. Finally, an experimental platform was built to test the performance of the sensor. The sensitivity of the gas sensor is about 0.04 MHz/ppm in an atmosphere with a NH_3_ concentration of 300 ppm. The sensor was shown to have good repeatability and high stability over a long time period.

## 1. Introduction

In recent years, various modern facilities have improved the convenience of daily life. At the same time, environment pollution has become increasingly serious [1,2,3]. As a result of the massive burning of coal and the arbitrary discharge of industrial waste gas, there are constant increases in air pollution [4]. How to effectively detect the composition and concentration of these gases in the surrounding environment has become a very important issue. Monitoring and studying such harmful gases, reducing the release of these gases and setting a threshold value are all very essential goals. Therefore, studies focusing on appropriate gas sensors are of great significance. Ammonia, which is a colorless gas with a pungent odor, is widely used in industry and agriculture [5,6]. It not only has a stimulating effect on human eyes, nose, and throat, but also denatures tissue proteins and destroys cell membranes [7,8]. Therefore, it is imperative that we have methods of ammonia gas detection.

Currently, wired sensors are mostly used in monitoring different gas concentrations, with one or more devices applied to identify different conditions of gas components and concentrations [9,10,11]. Such methods can only detect gas concentrations in extremely limited areas. If many gas sensors are used to form a large wired sensing system, this creates many problems, such as difficulty in wiring and high-power consumption as sensor node power needs to be replaced frequently [12,13]. In addition, a conventional semiconductor gas sensor consumes large amounts of energy because it requires a higher operating temperature [14]. The use of such sensors will significantly reduce the life span of the node. 

Deshpande et al. [15] reported that SnO_2_/PANI (polyaniline) nanomaterials detected ammonia at a concentration of 100–500 ppm at room temperature with a sensitivity of 3 ppm^−1^. Saxena [16] produced a polycarbazole LB film that operates at room temperature and has a linear relationship at an ammonia concentration of 300–5000 ppm with a sensitivity of 3 ppm^−1^. Airoudj et al. [17] reported that PANI was doped by plasma-enhanced chemical vapor deposition as measured by optical detection. They found that the sensitivity and the concentration of ammonia in the concentration range of 92–4618 ppm have a logarithmic linear relationship.

This article aims to achieve real-time gas monitoring in a closed environment at room temperature. Based on the principle of LC (Inductance and Capacitance) mutual coupling, the ADS (Advanced Design System) simulation software was used for simulating the designed sensor. The PANI/CNT (Polyaniline/Carbon Nanotube) composite with the advantages of simple preparation, cost-effectiveness and good stability can be used as a gas sensitive material to prepare a passive wireless gas sensor, which does not require a power supply internally. It has two natural advantages. First, there is no need to replace the battery and therefore, it has infinite life. Second, it can be used under certain special circumstances, such as a closed environment and a high temperature environment.

## 2. The Principle of Sensor

### 2.1. LC Resonant Circuit

A metal interdigitated structure is used as the sensor’s capacitor. This has a planar structure in which metallic conductors are placed in a comb-like arrangement [18].

The wireless passive gas sensor device designed in this paper realizes the wireless transmission of signals through electromagnetic coupling. In order to better understand the electromagnetic characteristics when the sensor and readout antenna are wirelessly coupled, it is regarded as being equivalent to a simple LC resonant circuit [19,20,21].

After the gas sensitive material is coated on the interdigital electrodes, this material becomes equivalent to a loose porous gas sensing layer. The sensing layer acts on the sensor by physically adsorbing gas and the relative dielectric constant *ε_r_* of the gas-sensitive layer will change. In turn, this will cause a change in the sensitive capacitance and eventually cause a change in the resonant frequency of the sensor [22].

**Figure 1:**
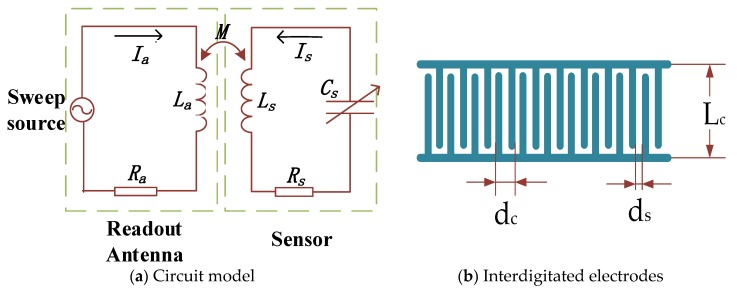
Schematic circuit model and interdigitated electrodes: (**a**) The circuit consists of a capacitor, an inductor and series resistance, where *R_a_* and *L_a_* respectively represent the equivalent resistance and equivalent inductance of the readout antenna; *L_s_*, *R_s_* and *C_s_* respectively represent the inductance, resistance and sensitive capacitance of the sensor circuit loop; and *M* represents the mutual inductance coupling coefficient between *L_a_* and *L_s_*. (**b**) *L_c_* is the length between the interdigitated electrodes; *d_c_* is the total spacing between two adjacent fingers; *d_s_* is the distance between two adjacent interdigitated fingers that are not covered by metal conductor; and *N_c_* is the number of interdigital pairs.

Referring to Figure 1, the sensor’s capacitance *C_s_* can be expressed as:

(1) $$C_{s}=L_{c}\left(N_{c}-1\right)\varepsilon_{0}\times \frac{1+\varepsilon_{r}K\left[{\left(1-{\left(d_{s}/d_{c}\right)}^{2}\right)}^{1/2}\right]}{2K\left(d_{s}/d_{c}\right)}$$

where *ε*_0_ is the vacuum dielectric constant and *K* is the first type of complete elliptic integral.

The resonance frequency *f*_0_ is given by [23]:

(2) $$f_{0}=\frac{1}{2\pi \sqrt{L_{s}C_{s}}}$$

From Equation (2), the change in the sensor’s resonant frequency *f*_0_ is related to the capacitance *C_s_* and the inductance *L_s_*. When a certain parameter in the closed environment changes, the capacitance or inductance in the LC resonant circuit would change. As a result, the sensor’s *f*_0_ will change correspondingly. The use of a network analyzer to detect the change in *f*_0_ indirectly indicates that a parameter has changed in an enclosed environment.

### 2.2. Gas Sensitive Infrastructure Simulation

**Figure 2:**
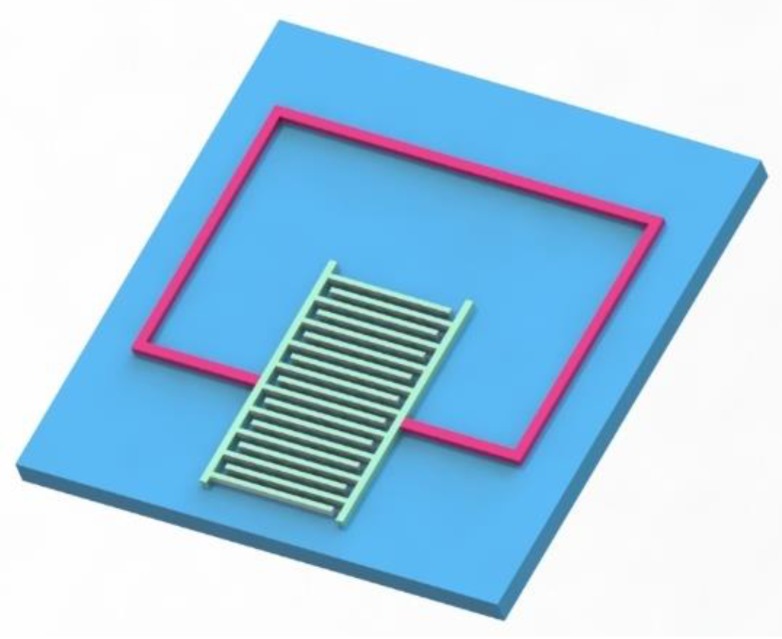
Schematic of sensor: The sensor designed in this article is based on a single-layer structure on a ceramic alumina substrate, with this special structure showing that the inductor and the interdigitated electrode can be printed together on the same surface.

In the capacitance of the planar metal interdigitated structure, the metallic conductors are placed in a comb-like arrangement. The structure of the sensor is shown in Figure 2. The gas-sensitive material can be directly applied in the interdigital capacitance.

**Figure 3:**
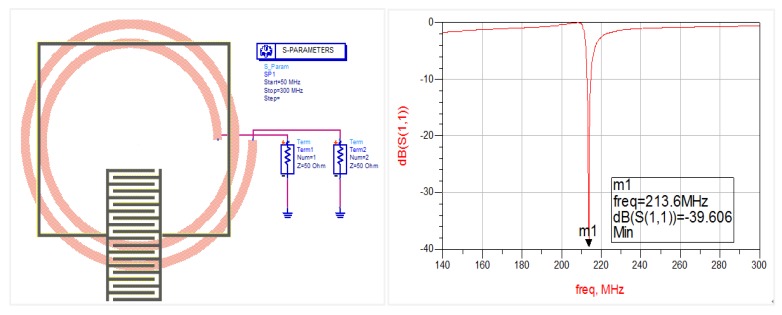
ADS simulation results over the bandwidth of 50–300 MHz, which is consistent with the range used experimentally. Resonance occurs when the frequency of the sweep signal is close to the natural frequency of the sensor at the minimum value of input return loss S (1,1). The theoretical resonant frequency of interdigital capacitor wireless passive gas sensor is found to be 213.6 MHz, with an input return loss of −39.61 dB.

The ADS software was used for simulating the interdigital capacitive infrastructure. The substrate material is alumina ceramic with a thickness of 2 mm and dielectric constant of 9. The conductive layer material is aluminum and the parameters are shown in Table 1. The thickness of the dielectric layer of SiO_2_ and PANI/CNT above is 100 μm, with the dielectric constant of SiO_2_ being 4.5, the distance between antenna and sensor being 5 mm and the dielectric constant of air being 1. The simulation results are shown in Figure 3.

## 3. Experiment Section

### 3.1. Preparation of PANI/CNT Composite

Since the sensitive properties of CNTs are often affected by some other impurities during the preparation process, the surface energy between the CNTs is large. Furthermore, the van der Waals forces between the CNTs cause the agglomeration of CNTs in many solutions, making it difficult to recombine them with other materials. In order to better combine the CNTs with other gas sensitive materials, the acidification of CNTs is performed to add functional groups. It not only increases the gas adsorption performance of CNTs, but also realizes the better mixing of CNTs with other sensitive materials.

The acidification of the carbon nanotubes was performed in a specific process. At room temperature, a mixed solution of concentrated H_2_SO_4_ and HNO_3_ with a volumetric ratio of 3:1 was prepared. After this, a certain amount of carbon nanotube powder was weighed (Chengdu Organic Chemistry Co., Ltd., Chinese Academy of Sciences, Chengdu, China), which was subsequently added to the mixed acid solution and soaked for 30 min, with the temperature maintained at 135 °C. After sufficient precipitation and cooling to room temperature, the supernatant was discarded and an appropriate amount of deionized water was added to the ultrasonic cleaner for 2 h. Carbon nanotubes were filtered out in a vacuum and repeatedly washed with deionized water until it became neutral. At this point, the CNTs were dried at 60 °C and we finally obtained acidified carbon nanotubes [24].

PANI/CNT composites were produced using in-situ polymerization. Aminobenzene was distilled under atmospheric pressure and stored at a low temperature for use. A total of 0.05 g of the chemically modified CNT was added to 45 mL of HCl acid solution (concentration: 1 mol/L), which was shaken by an ultrasonic cleaner for 1.5 h. A total of 2.5 mL of aminobenzene was distilled at atmospheric pressure, into which the CNTs were dispersed using a magnetic stirrer. After this, 50 mL (concentration: 0.1 mol/L) of (NH_4_)_2_S_2_O_8_ solution was added to the above mixed solution and finally, the temperature was maintained between 0 °C and 5 °C. This was subsequently placed in an ice-water bath for 24 h. The solution was filtered using a filtration device and washed repeatedly until the filtrate was colorless. Finally, the obtained composite PANI/CNT was dried in a 65 °C drying oven for 12 h. The preparation of pure PANI was similar to the above method, which was used as a reference group [25].

### 3.2. Characterization of Materials

#### 3.2.1. SEM Characterization

**Figure 4:**
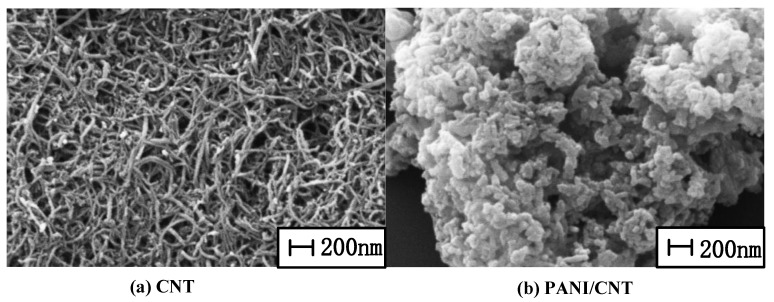
SEM image of two samples: (**a**) Unacidified CNT, in which other impurities exists with poor dispersion. (**b**) Polyaniline adhered to the surface of chemically acidified carbon nanotubes.

The sample was observed by scanning electron microscopy (SEM), as shown in Figure 4. Carbon nanotubes have a larger specific surface area and have good adsorption to different gas molecules. Since polyaniline is sensitive to ammonia molecules, the composite formed will be sensitive only to ammonia gas molecules and increase the ability of the sensitive material to adsorb ammonia gas molecules, thus increasing the sensor’s sensitivity in terms of a resonant frequency shift [26,27].

#### 3.2.2. Electrical Characterization

**Figure 5:**
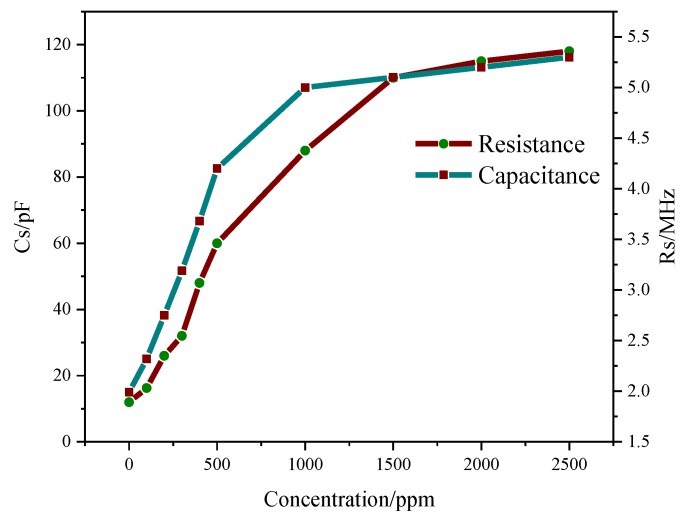
Capacitance and resistance experiments at different NH_3_ concentrations.

Figure 5 shows the measurements of capacitance and resistance of the sensor at different ammonia concentrations. The sensor was placed in a closed container, which was connected to the measuring instrumentation, and ammonia gas was introduced. It can be seen that the capacitance and resistance increase linearly at 0–1000 ppm and both gradually reach saturation as the concentration increases.

### 3.3. Gas Sensitivity Mechanism

**Figure 6:**
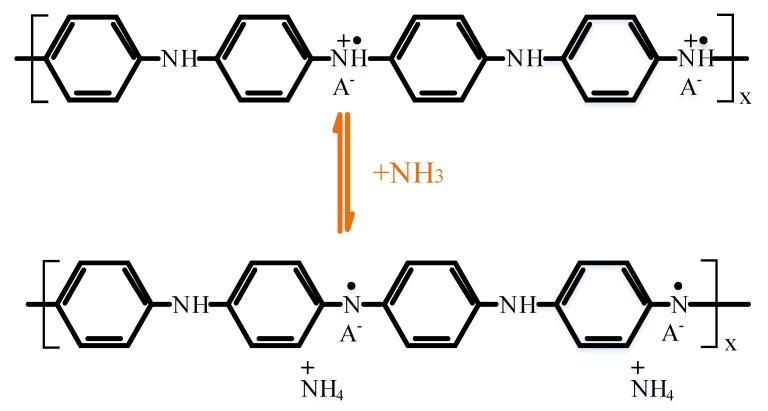
PANI sensitive mechanism to NH_3_: PANI exhibits the property of being a p-type semiconductor. When it comes into contact with an NH_3_ molecule with reductive ability, the NH_3_ molecule contains an unbonded valence electron and becomes an electron donor. As a result, the number of carriers in polyaniline decreases and the conductivity decreases, while NH_3_ molecules are adsorbed between the PANI chains and gas molecules enter the gas-sensing layer. The dielectric permittivity of the gas-sensing layer and gas molecules caused a change due to the larger specific surface area of CNT. PANI and CNT are doped to increase the ability of the sensitive material to adsorb gas molecules so that the gas molecules can adsorb more, which results in greater variation of the mixed dielectric constant and increased changes in sensor frequency.

The sensitivity mechanism of polyaniline to NH_3_ is shown in Figure 6. When PANI was mixed with NH_3_, the mixed dielectric constant *ε_r_* of the gas sensing layer increases, as shown by Equations (1) and (2). The sensor’s capacitance *C_s_* increases as *ε_r_* increases, while resonant frequency *f* decreases.

### 3.4. Preparation of LC Wireless Passive Gas Sensor

A lead-free aluminum paste was printed on an alumina ceramic substrate via the screen printing technique to form an inductor coil. First, the screen of the alumina ceramic substrate and the printed circuit board was fixed on the self-made fixed table, before the lead-free aluminum paste was uniformly placed on one side of the basic structure. The rubber scraper was evenly moved by the hand until the slurry covered the entire pattern. In this process, the direction of force should be consistent and the best angle is 45°. During the movement of the squeegee, the lead-free aluminum paste was deposited on the alumina ceramic substrate through the small holes in the screen, with the deposit thickness being about 25 μm.

The printed base structure was sintered at different high temperatures using a high-temperature energy-saving sintering furnace. The temperature rises to 200 °C in the first 40 min and was maintained for 20 min, which is intended to evaporate the liquid portion of the lead-free aluminum paste slurry so that it adheres better to the alumina ceramic substrate. After this, the temperature was increased to 700 °C and held for another 20 min. After the sintering is completed, the basic structure was taken out and cleaned with alcohol. Finally, the signal from the prepared sensor was detected.

**Figure 7:**
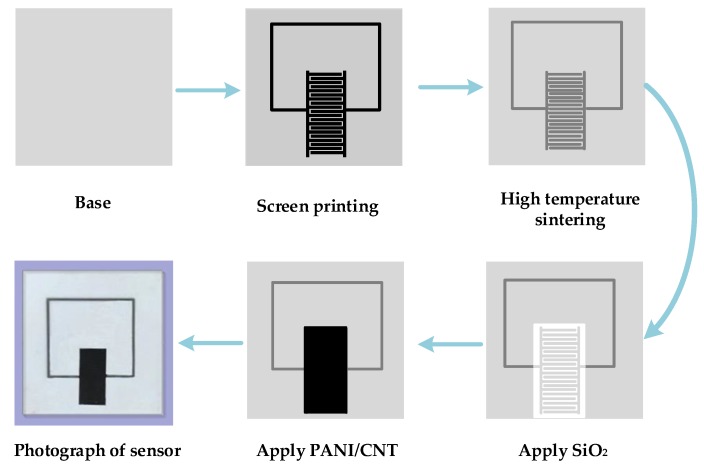
Process of sensor preparation.

Subsequently, an electrically insulating protective layer of SiO_2_ on the inductor was applied (because CNT and PANI are conductive) to avoid malfunctioning due to direct contact between the gas sensitive material and the inductor coil. After this, the well-tested gas sensitive material slurry was evenly coated with a SiO_2_ layer, with uniform smearing and thickness. Subsequently, it was allowed to cool to room temperature after being dried on a heating table at 150 °C for 2 h. A more stable structure of the sensor was formed, with the gas sensitive membrane being completely dry. An interdigital capacitive wireless passive gas sensor was obtained. The production process is shown in Figure 7.

### 3.5. Sensitivity Test of Gas Sensor

**Figure 8:**
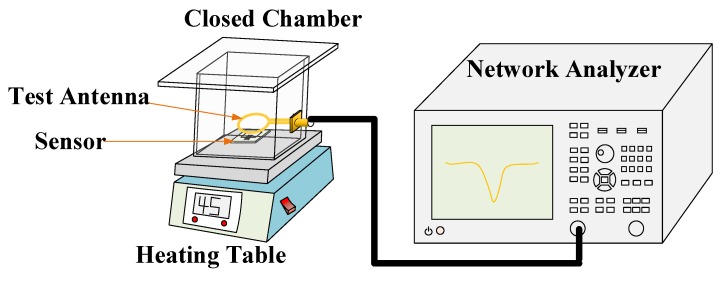
Schematic diagram of the test platform: It consists of a network analyzer, an external test antenna, a closed chamber and a heating station.

In this experiment, the ammonia gas with different concentrations of 100 ppm, 200 ppm, 300 ppm, 500 ppm, 1000 ppm, 1500 ppm, 2000 ppm and 2500 ppm was prepared. A total of 3000 ppm of ethanol gas and acetone gas was also prepared for gas specificity test. The test platform is shown in Figure 8. In order to verify the influence of temperature on the sensor, the sealed container was placed on a heating table and tested.

#### 3.5.1. The Relationship between the Resonant Frequency Variation and the Operating Temperature

The variation of general PANI gas sensors at different operating temperatures will be different since CNT has a special structure due to being composed of a nanomaterials and polyaniline composite, which greatly improves the degree of gas adsorption. A stronger absorption capacity increases the amount of adsorbed gas, which results in an increase in the dielectric constant of the gas-sensitive material and an increase in the capacitance. The resonant frequency increased according to Equation (2). Table 2 shows the variation in the resonant frequency of PANI and PANI/CNT sensors at different temperatures in a 300-ppm ammonia atmosphere. It is evident from the table that the resonant frequency variation of PANI/CNT gas sensor has been effectively improved and that the maximum change at 45 °C is 12.07 MHz. Furthermore, the variation of the resonant frequency of a pure PANI gas sensor is not large as the maximum change is only 2.857 MHz. It can be concluded that the PANI/CNT sensor has a larger adsorption capacity than the pure PANI sensor and the optimal operating temperature is 45 °C.

#### 3.5.2. Repeatability Test

**Figure 9:**
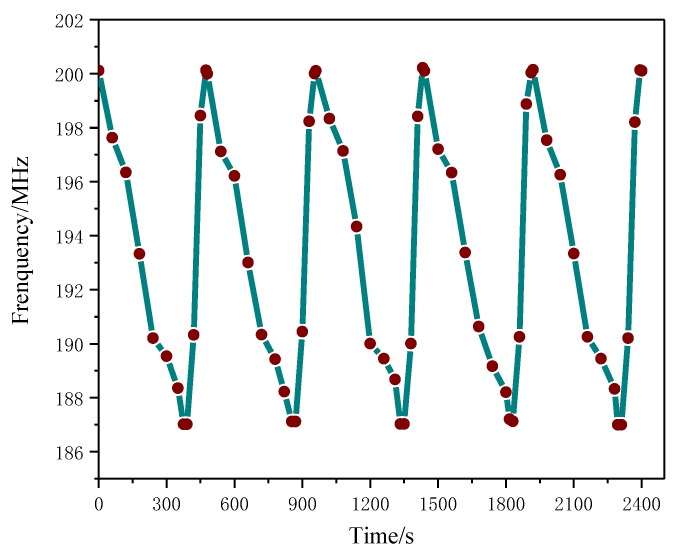
Gas adsorption and desorption time curves.

Figure 9 shows the adsorption and desorption time curve of NH_3_ on a PANI/CNT sensor at an optimal temperature of 45 °C in an atmosphere of 300 ppm. It can be seen from the figure that the natural frequency of the sensor coated with the gas-sensitive material is 200 MHz, which is lower than the ADS simulated resonant frequency. This is mainly because the actual losses of the real materials and the electrical properties of the sensitive layer (PANI/CNTs) were not taken into consideration. After the gas was introduced, the resonant frequency changed by 12 MHz. The lowest value is 188 MHz with a response time of about 375 s. After this, the lid of the sealed container was removed; the sensor and the surrounding air were in full contact with each other; and the NH_3_ gas molecules began to be desorbed. The frequency gradually returned to its starting value with a recovery time of about 84 s. The corresponding results of 5-time repeated tests are approximately the same, indicating a good repeatability of the sensor.

#### 3.5.3. Gas Sensing Properties of Composite

**Figure 10:**
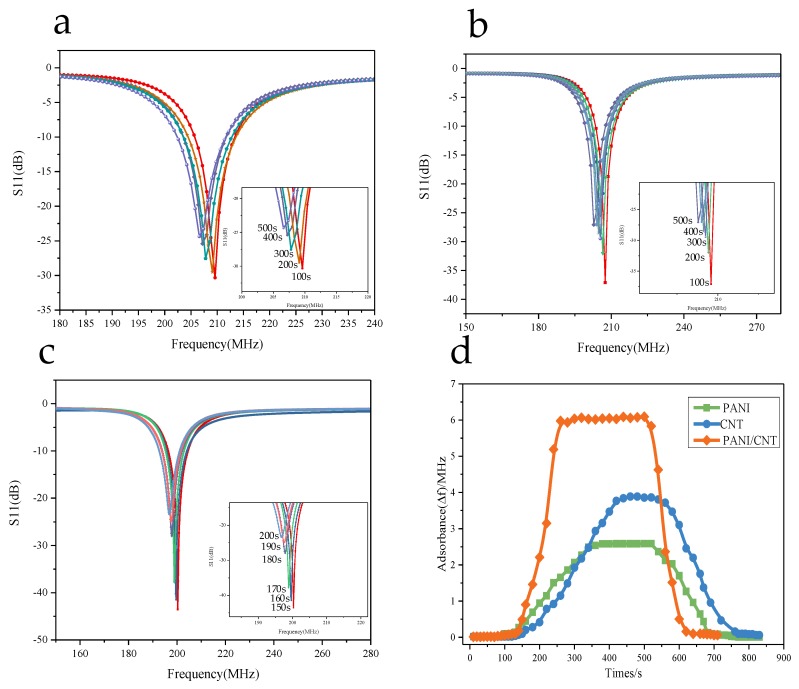
Adsorption performance of different materials for ammonia: (**a**) The data when PANI adsorbs ammonia to the stable state, with the maximum frequency variation being 2.5 MHz; (**b**) The data when CNT adsorbs ammonia to the stable state, with the maximum frequency variation being 3.9 MHz; (**c**) The data when PANI/CNT adsorbs ammonia to the stable state, with the maximum frequency variation being 6 MHz; (**d**) Adsorption capacity and response recovery time diagram. By comparison, we found that the sensor coated with PANI/CNT composites has better adsorption performance than the other two sensors, with the time for the adsorption and desorption of NH_3_ gas molecules by the sensor being significantly shortened.

Three identical sensors were prepared, before three materials, which were namely PANI, CNT and PANI/CNT, were sprayed onto the sensors. The sensor was placed in a closed container, as shown in Figure 8, with the distance between the sensor and test antenna maintained at approximately 5 mm. At room temperature, the antenna was connected to the network analyzer and data were recorded every 10 s after the injection of ammonia, which was stopped when the ammonia concentration reached 300 ppm. The experimental data are shown in Figure 10.

#### 3.5.4. Relationship between Gas Concentration and Resonant Frequency

**Figure 11:**
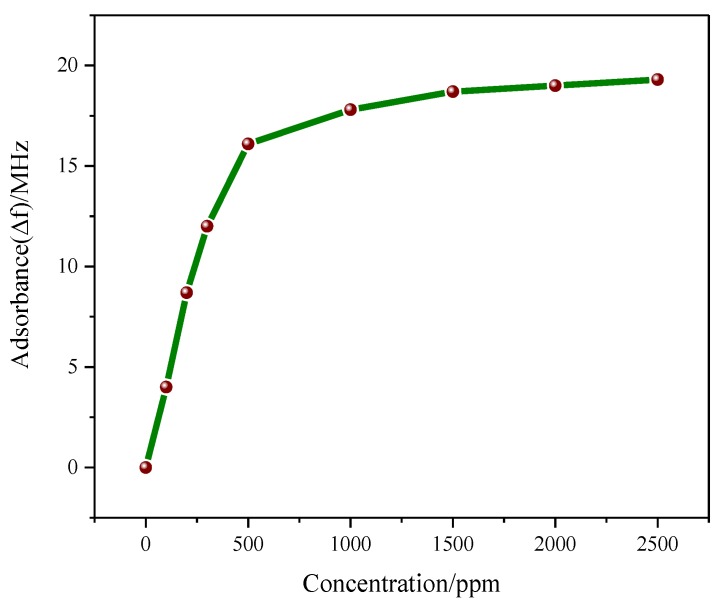
The relationship between ∆*f* and concentration: In the range of 0–500 ppm, the change in resonant frequency shows a linear growth trend. When the concentration of NH_3_ reaches 500 ppm or above, the change in resonant frequency remains basically same with any further increase in the concentration of NH_3_, indicating that when the concentration of NH_3_ reaches a certain value, the ability of the sensor to adsorb NH_3_ molecules reaches a steady state.

Figure 11 shows the graph of the change in resonant frequency in 0–2500 ppm NH_3_ atmosphere at an optimal temperature of 45 °C. It can be seen that the gas sensor can detect a wide range of NH_3_ concentrations.

**Figure 12:**
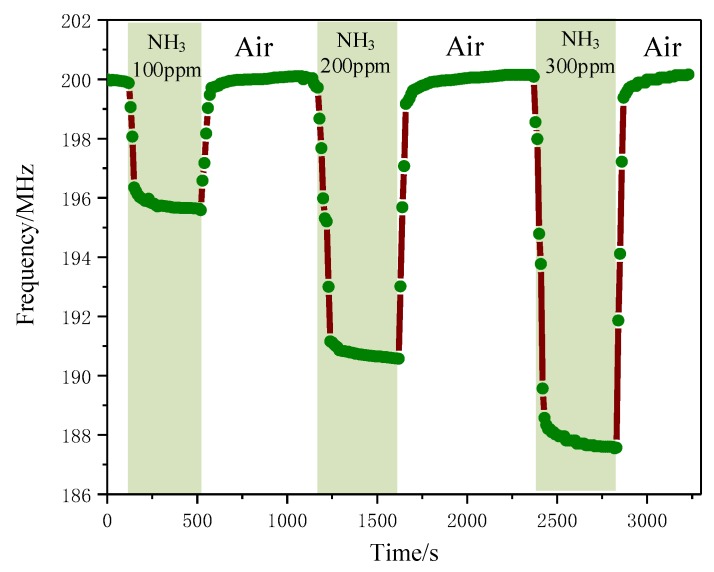
Response and recovery time curve at different concentrations: It can be seen that the variation of *f*_0_ is 4.352 MHz, 9.235 MHz and 12.070 MHz, respectively; the sensitivity of gas sensor is about 0.04 MHz/ppm under the concentration of 300 ppm. With a low concentration, the variation of resonant frequency is relatively large.

Figure 12 shows the response and recovery time profiles of PANI/CNT sensors in three different concentrations of NH_3_, which were namely 100, 200 and 300 ppm. The experiments concerning the adsorption and desorption were performed under different concentrations of NH_3_. All properties of the sensor were relatively stable, indicating that the gas sensor has good stability.

#### 3.5.5. Sensor Specificity and Long-Term Stability

**Figure 13:**
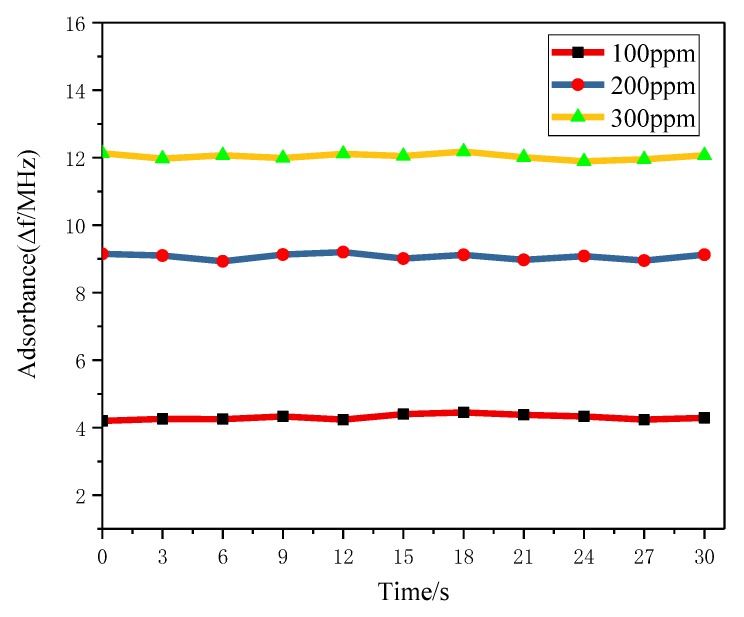
Sensor Stability: It can be seen that the sensor’s resonant frequency in long-term operation and the amount of change remain basically unchanged, indicating that the sensor has good stability.

Figure 13 shows the time variation curve of the gas sensor at an optimal temperature of 45 °C in NH_3_ with gas concentrations of 100 ppm, 200 ppm and 300 ppm.

**Figure 14:**
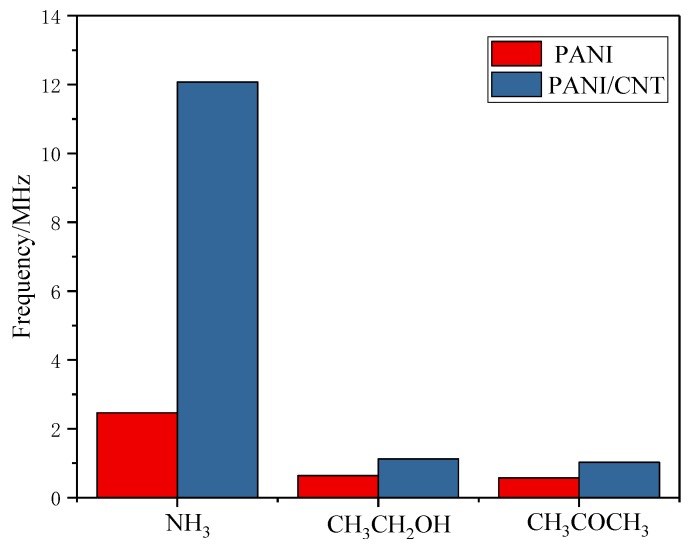
Variation of *f*_0_ of different gases: It can be seen that in different gases, the variation of the PANI gas sensor is lower than that of PANI/CNT-related one In the 300-ppm NH_3_ atmosphere, the variation of the resonant frequency of the PANI/CNT sensor reaches 12.070 MHz, which is 5.4 times as that of C_2_H_5_OH and 5.9 times of CH_3_COCH_3_.

Figure 14 is the graph regarding the variation of the resonant frequency in the atmosphere of 300 ppm NH_3_ and 3000 ppm C_2_H_5_OH and CH_3_COCH_3_ in the PANI and PANI/CNT sensors at a working temperature of 45 °C.

## 4. Conclusions

In this paper, a wireless passive gas sensor based on alumina ceramic was designed. The acidified CNT and PANI composites were used as a gas sensitive material. The gas sensing layer acts on the sensor by physically adsorbing NH_3_ molecules. On the other hand, the wireless passive measurement was realized through the changes in resonant frequency. The relevant tests showed that the sensor can detect a wide range of concentration with a sensitivity of 0.04 MHz/ppm in a 300-ppm NH_3_ atmosphere. The sensor showed better specificity for NH_3_ when comparing the gases of NH_3_, C_2_H_5_OH and CH_3_COCH_3_. Repeatability tests showed that the sensor has good repeatability. The advantage of this sensor is that there is only one inductor coil with no influence of parasitic capacitance existing between the coils. Only the change in resonant frequency occurred due to the capacitance change between the fingers.

## Figures and Tables

**Table 1:** Various parameters of sensor.

Parameter		Value
Number of finger pairs	*N_c_*	10
Finger width	*L_c_*	6 mm
Gap between fingers	*d_c_*	2 mm
Gap at ends of fingers	*d_s_*	0.5 mm
Thickness of metal films	*t*	25 μm
Number of inductor coil	*N*	1

**Table 2:** Relationship between *f*_0_ variation and temperature.

	*f*_0_/MHz	PANI	PANI/CNT
Temperature/°C			
25		1.5	6.7
35		2.2	9.8
45		2.857	12.07
55		2.94	10.68
